# Platelet activation risk index as a prognostic thrombosis indicator

**DOI:** 10.1038/srep30508

**Published:** 2016-07-27

**Authors:** K. E. Zlobina, G. Th. Guria

**Affiliations:** 1 National Research Center for Hematology, 125167, Novy Zykovsky pr. 4, Moscow, Russia; 2 Moscow Institute of Physics and Technology, 141700, Institututski per. 9, Dolgoprudny, Russia

## Abstract

Platelet activation in blood flow under high, overcritical shear rates is initiated by Von Willebrand factor. Despite the large amount of experimental data that have been obtained, the value of the critical shear rate, above which von Willebrand factor starts to activate platelets, is still controversial. Here, we recommend a theoretical approach to elucidate how the critical blood shear rate is dependent on von Willebrand factor size. We derived a diagram of platelet activation according to the shear rate and von Willebrand factor multimer size. We succeeded in deriving an explicit formula for the dependence of the critical shear rate on von Willebrand factor molecule size. The platelet activation risk index was introduced. This index is dependent on the flow conditions, number of monomers in von Willebrand factor, and platelet sensitivity. Probable medical applications of the platelet activation risk index as a universal prognostic index are discussed.

In many cases, intravascular blood coagulation, followed by myocardial infarctions and strokes, is known to be the result of rapid increase in arterial pressure and relevant hemodynamic characteristics such as blood velocity and flow shear rate^1^,^2^. However, modern clinical tests are limited to predict thrombotic or bleeding risk because they measure clotting behavior under static (no flow) conditions^3^. Only first steps are made towards development of diagnostic methods and devices for measuring effects of hemodynamic forces that contribute to platelet function and thrombus formation^4^,^5^.

Von Willebrand factor (VWF) plays a central role in transmitting the dynamic effect of blood flow shear stress to intracellular platelet activation pathways^6^,^7^,^8^,^9^,^10^,^11^,^12^,^13^. VWF has a multimer structure, consisting of 2–80 monomers per multimer in the blood of healthy donors^14^,^15^. Each monomer contains an A1 domain that is capable of binding to the platelet receptor GP-Ib, thereby initiating platelet activation. VWF is present in blood in the globular form. Following an increase in shear rate, it unfolds into an elongated form, which has an increased binding ability^16^,^17^ (Fig. 1).

It is known that platelet activation by VWF in a shear flow occurs only at high, overcritical shear rates. However, until recently, the value of the critical shear rate has been controversial, despite the presence of numerous experimental data^8^,^12^,^17^,^18^,^19^. It is well established^7^,^8^,^20^,^21^ that the value of the critical shear rate 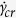 is between 1000 sec^−1^ and 10000 sec^−1^.

The presence of large VWF multimers in blood causes thrombotic disorders^18^,^22^,^23^,^24^, while the decreased size of VWF multimers is known to be a factor in bleeding disorders^25^,^26^. The actual dependence of the critical shear rate value 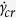 on VWF molecule size has not yet been discussed.

## Von Willebrand factor unfolding

**Figure 1:**
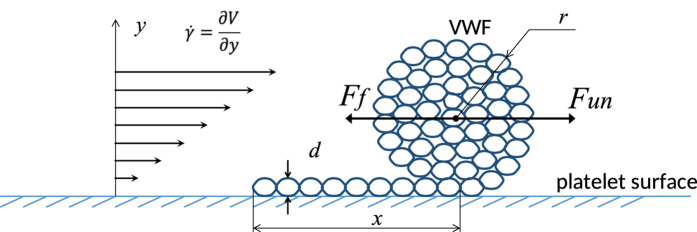
Partially unfolded VWF molecule globule in shear flow, *r* denotes the radius of the globular part, *x* is a length of the unfolded “tail”. Diameter of a single chain of multimer is denoted as *d*. Blood shear rate is denoted as 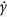
*. F*_*f*_ is the folding force and *F*_*un*_ is the unfolding force.

A VWF multimer grafted onto a platelet surface in shear flow (Fig. 1) is exposed to at least two forces. The first force, *F*_*un*_, is induced by the ongoing blood flow and unwinds the multimer from the globular to the stretched form^17^,^27^,^28^,^29^. The second force, *F*_*f*_, is derived from the “effective surface tension,” which tends to wind the multimer into the most compact globular form. Both of these forces are dependent on the multimer size and its conformation.


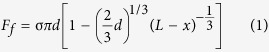



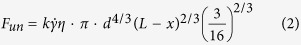


where σ corresponds to the effective “surface tension”, *d* refers to the diameter of VWF monomeric subunits, *L* denotes the total length of the VWF molecule, *x* denotes the length of the “tail” (the unwound part of the molecule), 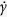 is shear rate, *η* reflects the value of blood viscosity, *k* is a dimensionless proportionality coefficient.

A detailed mathematical model of VWF multimer folding-unfolding is presented in the Supplementary information. An analysis of the equilibrium conditions between the folding and unfolding forces revealed three states of VWF grafting on platelets:

• fully folded globular structure;

• partially folded globule with an unfolded “tail”;

• fully unfolded linear structure.

**Figure 2:**
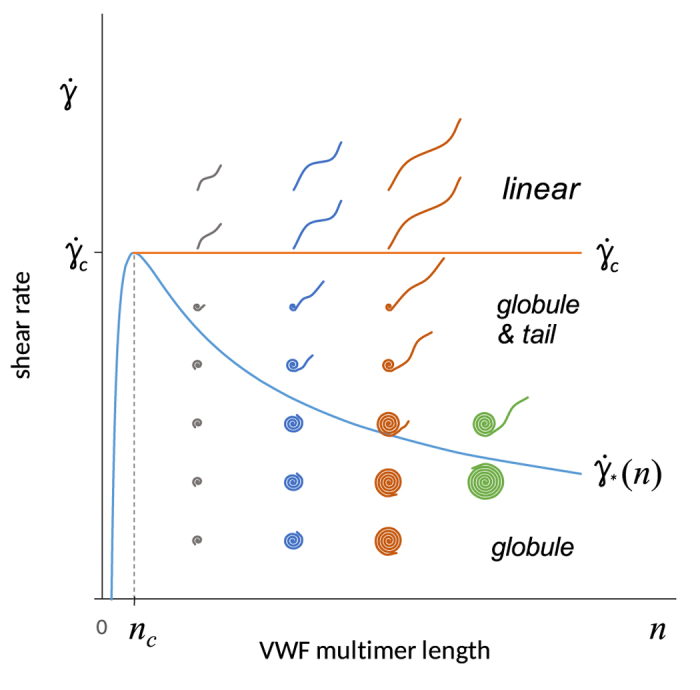
Parametric diagram of VWF folding-unfolding. Three domains corresponding to a globule, to a globule with tail and to a linear state of VWF multimer are demonstrated. The relative size of VWF globular and linear parts is shown qualitatively in each domain.

The existence and stability of each of these states are dependent on the VWF multimer size (*n*, monomers per multimer) and shear rate 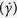. The parametric plane is presented in Fig. 2.

The condition of the fully folded “globule” state is described by the inequality:


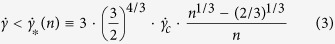


(see the Supplementary information).

The curve 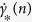 reaches maximum at *n* = *n*_*c*_ = 9/4. The model is hardly applicable to very short multimers. Further we discuss only the case *n* > *n*_*c*_.

The curve 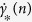 monotonlically decreases, and the transition from a globule to a partially folded globule with a tail occurs at lower shear rates for larger multimers.

**Figure 3:**
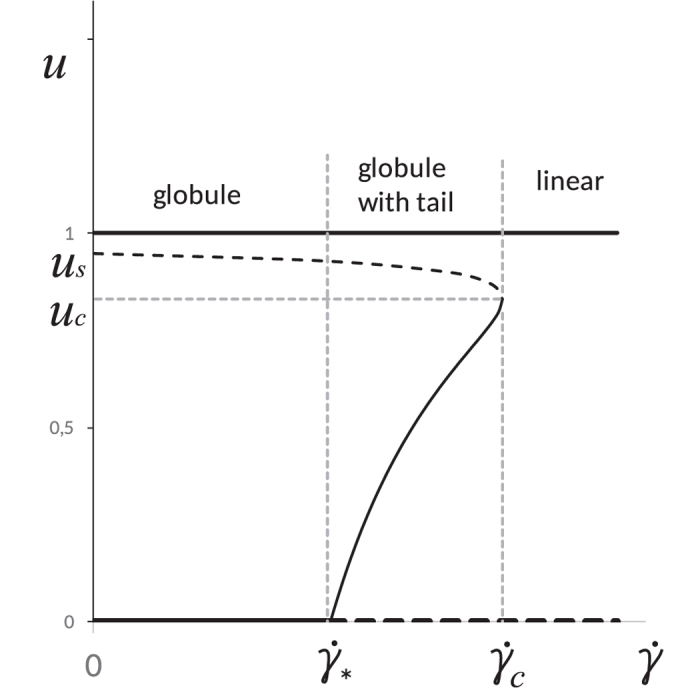
Bifurcation diagram of VWF multimer for *n* > *n*_*c*_. “*u*” denotes a relation of number of monomers in the unwound tail to the total number of monomers in VWF molecule (*u* = *n*_*tail*_/*n* ≡ *x*/(*nd*)). Branches relevant to stable stationary states are shown by solid line while branches relevant to unstable states – by dashed line.

At any fixed value of *n*, a gradual increase in 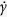 is followed by several conformational changes in the VWF structure. This is demonstrated in the bifurcation diagram presented in Fig. 3, where *u* is the relative length of the unfolded VWF tail.

At low shear rates 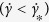, a VWF multimer is a fully folded globule. If the shear rate increases above 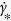, the multimer begins to unfold, becoming a globule with a tail. Further increases in the shear rate above 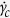, results in the unwinding of all VWF multimers regardless of their size.

## Platelet activation

In this study, we assumed that only an unfolded VWF multimer exposing a sufficient amount of A1 domains can activate platelets via simultaneous binding to a group of GP-Ib receptors in an “accord” manner. In other words, we assumed the existence of “minimal platelet activation accord” (*n*_*A*_), i.e., a minimal amount of A1 domains on the unfolded part of VWF, which is sufficient for platelet activation.

**Figure 4:**
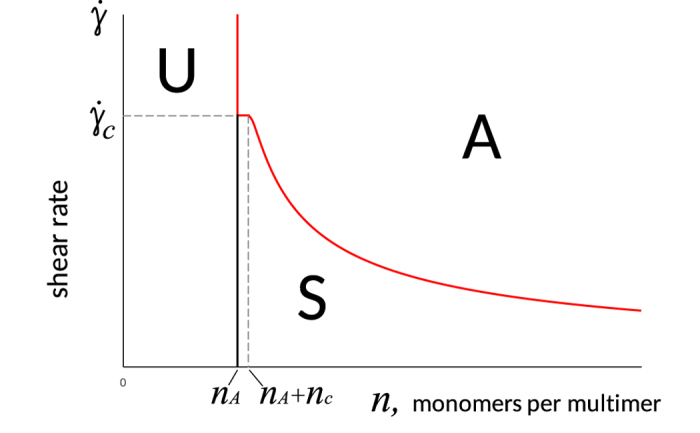
Platelet activation diagram. Solid lines divide the plane into three domains. Domain U corresponds to parameters at which platelets cannot be activated by VWF (*n* < *n*_*A*_). Domain A is relevant to parameters at which the length of the VWF tail is sufficient for platelets activation. Domain S refers to subcritical states (*n*_*tail*_ < *n*_*A*_), but platelet may be eventually activated. The curve between domains “S” and “A” is described by Eq. (4).

In Fig. 4, a platelet activation diagram is presented. The area “U” corresponds to short VWF multimers containing less than the critical number of monomers per multimer, *n* < *n*_*A*_. VWF is unable to activate platelets.

Domains “S” and “A” correspond to larger VWF multimers, *n* > *n*_*A*_. In domain “A,” the unfolded part of the VWF multimer (i.e., the “tail” in the case of a “globule-with-tail” or the full multimer in the case of a fully unfolded multimer) contains more than *n*_*A*_ monomers. In domain “S,” the number of monomers in the unfolded part of VWF is less than *n*_*A*_. In other words, domain “A” corresponds to the conditions required for platelet activation, while “S” domain corresponds to “subcritical” platelet activation and “U” corresponds to non-activation conditions.

The mathematical expression for the value of the critical shear rate 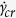 sufficient for platelet activation has the form:


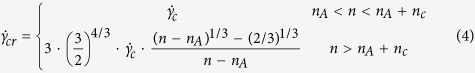


(see the Supplementary information).

The necessary and sufficient condition for platelet activation, 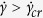, is equivalent to 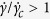 for a small interval of VWF multimer sizes, 0 < *n* − *n*_*A*_ < *n*_*c*_, and to:


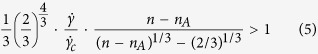


for *n* − *n*_*A*_ > *n*_*c*_. We denote the left part of this expression as the “platelet activation risk index” (PARI).

Taking in mind that in reality shear-induced platelet activation is governed by the value of shear stress (*τ*) rather than shear rate^20^ 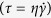 for practical purposes, the following equation may be used instead of (5):


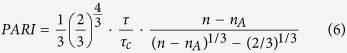


where 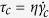 and *η* is viscosity.

## Discussion

To the best of our knowledge, the hypothesis that effective surface tension plays an important role in VWF folding-unfolding dynamics has not yet been analyzed. However, in polymer physics, a similar approach for the determination of the degree of polymer unwinding was developed at the end of the 20^th^ century^28^,^29^.

By applying the approach developed by De Gennes^28^ and his colleagues^29^ to the VWF dynamics under shear stress conditions on the surface of platelets, we obtained for the first time the exact expression (4) for the dependence of the critical shear rate of platelet activation on VWF multimer size.

An essential assumption introduced in the present work concerns the concept of minimal platelet activation accord. This concept involves the clustering of GPIb receptors on the platelet surface in shear flow, which should enhance platelet interaction with VWF^30^. In this study, we propose that the clustering and cooperative action of platelet GPIb receptors occurred due to their binding to a sufficient amount of A1 domains on the VWF multimer.

Many researchers have observed that long VWF multimers activate platelets more easily while shorter molecules demonstrate a decreased ability to activate platelets or even a lack of ability^18^,^22^,^23^. Recently, it was shown that VWF multimers of less than 5500 kDa (22 monomeric units) cannot induce platelet activation^23^. Under the experimental conditions described, *n*_*A*_ = 22. It is known that the ability of platelets to be activated by VWF varies among different platelet subpopulations^31^. The dependence of *n*_*A*_ on various pharmaceuticals is of great interest.

VWF multimers are dispersed by size in blood. There is a wide range of hydrodynamic conditions in the blood circulation^32^. Thus, when trying to apply our results to real situations, instead of a specific representation point on the diagrams (Figs 2 and 4), corresponding representation clouds should be considered. The fraction of the representation cloud located in domain “A” on the platelet activation diagram (Fig. 4) should qualitatively reflect the integral intensity of platelet activation.

To easily quantify platelet activation, we introduced the PARI. If PARI < 1, platelets are not activated. If PARI > 1, platelets are activated by VWF. The PARI is dependent on VWF size (*n*), shear rate 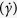, and platelet sensitivity (*n*_*A*_). The value of 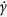 may be calculated mathematically^33^ or directly determined (by MRI or ultrasonic data). The procedure used to determine the VWF size distribution is also known^34^.

There are several methods of regulating the value of PARI, including varying the arterial pressure and vessel geometry or controlling the distribution of VWF size, *n*.

VWF size is regulated by ADAMTS13, which cuts VWF into shorter multimer fragments^33^. An enhanced shortening of VWF multimers by ADAMTS13 is known as von Willebrand disease 2A^25^,^26^ (decreased platelet activation), and it corresponds to the shifting of representative points to the left in the platelet activation diagram (Fig. 4) and to a decreasing PARI.

The ADAMTS13-related increase in VWF length^35^ is known as thrombotic thrombocytopenic purpura and corresponds to a shifting of the representation cloud to the right in Fig. 4, resulting in easier platelet activation and a tendency toward thrombosis. Additionally, the value of the PARI is increased.

In this study, we found that the value of the critical shear rate of platelet activation is dependent on VWF multimer size. An integrative PARI was introduced. Correction of the PARI may be accomplished by the regulation of system hemodynamics, by a pharmacological change in platelet sensitivity, or by the regulation of VWF distribution by size. The PARI may be used as a universal prognostic index.

## Additional Information

**How to cite this article**: Zlobina, K. E. and Guria, G. Th. Platelet activation risk index as a prognostic thrombosis indicator. *Sci. Rep.* **6**, 30508; doi: 10.1038/srep30508 (2016).

## Supplementary Material

Supplementary Information
