# Supplementary material for: Platelet activation risk index as a prognostic thrombosis indicator
Source: Sci Rep. 2016 Jul 27;6:30508. doi: 10.1038/srep30508 (PMC4962318; doi:10.1038/srep30508)
Supplement: Supplementary Information [file srep30508-s1.pdf]

Supplementary Information for:

Platelet activation risk index as a prognostic thrombosis indicator

K.E. Zlobina, G.Th. Guria
correspondence to: guria@blood.ru

In this work we assume that VWF is packed into a dense globular-like structure when no hydrodynamic tension is applied. We only consider the case of a VWF grafted on the surface of a platelet. There are two types of forces acting on the grafted macromolecule in blood under flow conditions. The first one is the so-called drag-force arising due to the action of blood movement. The second one is caused by "effective surface tension" tending to transform the macromolecule to the most compact form – to a globule (see Fig. 1).

Using the simplest approximation[1,2] the drag-force may be estimated as:

$$F_{un} = k\dot{\gamma}\eta \cdot \pi r^2 \quad \text{(S1)}$$

where r denotes the radius of the globule, $\dot{\gamma}$ corresponds to the shear rate and η reflects the value of blood viscosity, k is a dimensionless proportionality coefficient. We consider only the drag force acting on the globule, supposing that it is larger than the drag force acting on the linear part of the multimer. The drag force is denoted as F_{un}, where the subscript "*un*" alludes to "unfolding".

To define the folding force F_f, consider the energy of VWF molecule interaction with the surrounding blood. This energy is proportional to the VWF molecule surface:

$$E = \sigma(\pi x d + 4\pi r^2) \quad \text{(S2)}$$

where σ corresponds to the effective "surface tension", x denotes the length of the "tail" (the unwound part of the molecule) and d refers to the diameter of VWF monomeric subunits. The absolute value of the folding force is defined by first derivative of E by x:

$$\frac{\partial E}{\partial x} = \frac{\partial}{\partial x}[\sigma(\pi d x + 4\pi r^2)] \quad \text{(S3)}$$

The surface tension forces are supposed to be prevailing over other folding forces (hydrogen bonds and other interactions). Due to the fact that the total volume of VWF remains unchanged in any winding-unwinding processes the following expression is valid:

$$L\frac{\pi d^2}{4} = x\frac{\pi d^2}{4} + \frac{4}{3}\pi r^3 \quad \text{(S4)}$$

where L denotes the total length of the VWF molecule.

The relation may be rewritten in the following form:

$$r = \sqrt[3]{\frac{3}{16}d^2(L-x)} \quad \text{(S5)}$$

Keeping this in mind for the absolute values of folding and unfolding forces one has:

$$F_f = \sigma\pi d\left[1 - \left(\frac{2}{3}d\right)^{1/3}(L-x)^{-\frac{1}{3}}\right] \quad \text{(S6)}$$

$$F_{un} = k\dot{\gamma}\eta \cdot \pi \cdot d^{4/3}(L-x)^{2/3}\left(\frac{3}{16}\right)^{2/3} \quad \text{(S7)}$$

Thus the balance between the folding and unfolding forces takes place when an equality:

$$F_f = F_{un} \quad \text{(S8)}$$

is satisfied.

This expression may be written in a dimensionless form:

$$1 - \xi^{-1/3}(1-u)^{-1/3} = \tilde{\gamma}\xi^{2/3}(1-u)^{2/3} \quad \text{(S9)}$$

where a new variable $u = x/L$ corresponding to the portion of the multimer chain in the unfolded tail and two dimensionless parameters: $\tilde{\gamma} = k\dot{\gamma}\eta d/4\sigma$ proportional to the shear rate and $\xi = 3L/2d$ proportional to the total VWF multimer length, –are introduced.

The left-hand side of eq. (S9) represents the dimensionless folding force: $f_f = \frac{F_f}{\sigma\pi d}$ while the right-hand side represents the unfolding force: $f_{un} = \frac{F_{un}}{\sigma\pi d}$.

Below, it will be demonstrated that under certain conditions the equality (S9) is not satisfied. In the case when $f_{un}>f_f$ the molecule will evolve to a more unwound state, while in the case when $f_{un}<f_f$, – to more wound state.

Bifurcation analysis

Consider the expression (S9). It is clear that if at some moment the right-hand side is greater than the left-hand side, then the unfolding force prevails and the globule should unfold. This remains true until both forces become equal to each other or until the globule becomes a fully unfolded chain, i.e. u=1 is satisfied.

On the contrary, if the left-hand side of (S9) is greater than the right-hand one, then the folding force predominates and the chain should fold. This remains true until folding and unfolding forces become equal or until the chain fully folds into a globule, i.e. u=0 is satisfied.

It is easy to establish that in principle, only three types of collocation of plots $f_{un}(u)$ and $f_f(u)$ are possible. All of them are shown in Fig. S1, where the plots corresponding to the dependence of the folding force on u are shown with dash-dot lines while the plots relevant to the unfolding force – with solid lines.

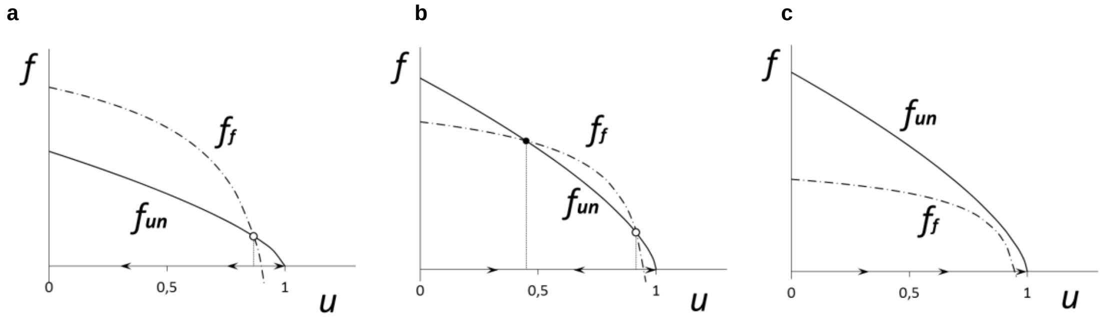

Fig. S1. Plots of folding (dash-dot line) and unfolding (solid line) forces. Three possible types of collocations of $f_f(u)$ and $f_{un}(u)$ are presented. The arrows on the abscissa axis indicate the direction of the variable u changing: u grows if $f_{un}>f_f$ and it decreases if $f_{un}<f_f$.

If the relation between the folding and unfolding forces is as shown in Fig. S1a, then steady states corresponding to fully folded (u=0) and a fully unfolded (u=1) VWF molecule are stable with respect to finite amplitude perturbations, while intersection of the plots in Fig. S1a corresponding to a partially unfolded molecule is absolutely unstable. Indeed, the folding force prevails on the left side of the intersection point (see Fig. S1a) and the unfolding force prevails on the right side of it.

If the plots relevant to folding and unfolding forces have two intersections as demonstrated in Fig. S1b, then the right intersection corresponds to an unstable partially unfolded state, and the left intersection of plots corresponds to a state stable with respect to finite amplitude perturbations. The fully unfolded state (u=1) is locally

stable. The state u=0 is unstable, that means that at certain values of parameters the globule cannot stay in a fully folded state.

Figure S1c corresponds to the situations when the plots of folding and unfolding forces have no intersections. Unfolding force always prevails that means that the fully unfolded state u=1 is stable and the fully folded state u=0 is absolutely unstable.

It is worth mention that no other variants of the relative location of plots $f_{un}(u)$ and $f_f(u)$ exist. In principle, the list of situations considered above is complete.

Based on the analysis presented above, a parametric plane $(\xi, \tilde{\gamma})$ can be divided into 3 domains (see Fig. S2). Three domains denoted as "globule", "globule with tail" and "linear" correspond to the three different types of steady states. Domain "globule" corresponds to the situation relevant to Fig. S1a, domain "globule with tail" to Fig. S1b and domain "linear" to Fig. S1c.

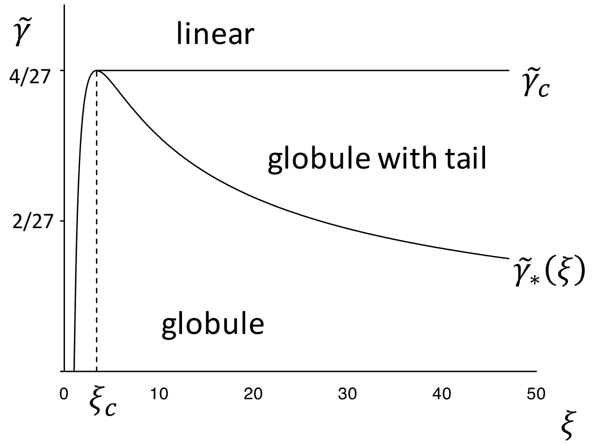

Fig. S2. Parametric diagram of stability of VWF molecules. The plane of parameters $(\xi, \tilde{\gamma})$ is divided into 3 domains: "globule" corresponds to one intersection of $f_f(u)$ and $f_{un}(u)$ plots as shown in Fig. S1a; "globule with tail" corresponds to Fig.S1b; "linear" corresponds to Fig. S1c. $\tilde{\gamma}$ denotes the dimensionless shear rate ($\tilde{\gamma} \equiv k\dot{\gamma}\eta d/4\sigma$), ξ denotes the dimensionless total length of a VWF multimer ($\xi \equiv 3L/2d$), $\xi_c = 27/8$.

It is worth mentioning that under the values of the parameters from domains "globule" and "globule with tail", a fully unfolded state also exists.

Solid lines restricting the domains in Fig. S2 correspond to bifurcations taking place in VWF molecules under variations of parameters. Domain "globule" is bounded by the curve $\tilde{\gamma} = \tilde{\gamma}_*(\xi)$:

$$\tilde{\gamma}_*(\xi) = \frac{\xi^{1/3} - 1}{\xi} \quad \text{(S10)}$$

Strait line $\tilde{\gamma} = \tilde{\gamma}_c$ separating the domains "globule with tail" and "linear" is defined by the expression:

$$\tilde{\gamma}_c(\xi) = \frac{4}{27}, \quad \text{for} \quad \xi > \xi_c = 27/8 \quad \text{(S11)}$$

Bifurcation diagrams of the system under consideration are shown in Fig. S3, where the dimensionless shear rate $\tilde{\gamma}$ is used as a bifurcation parameter. It is obvious that there are two different types of transitions from the folded ("globule") to the unfolded ("linear") state. The first type implies a direct transition that takes place at the values of parameter ξ less than ξ_c. This transition seems to be classified as a far from equilibrium first-order transition (see Fig. S3a)[3,4].

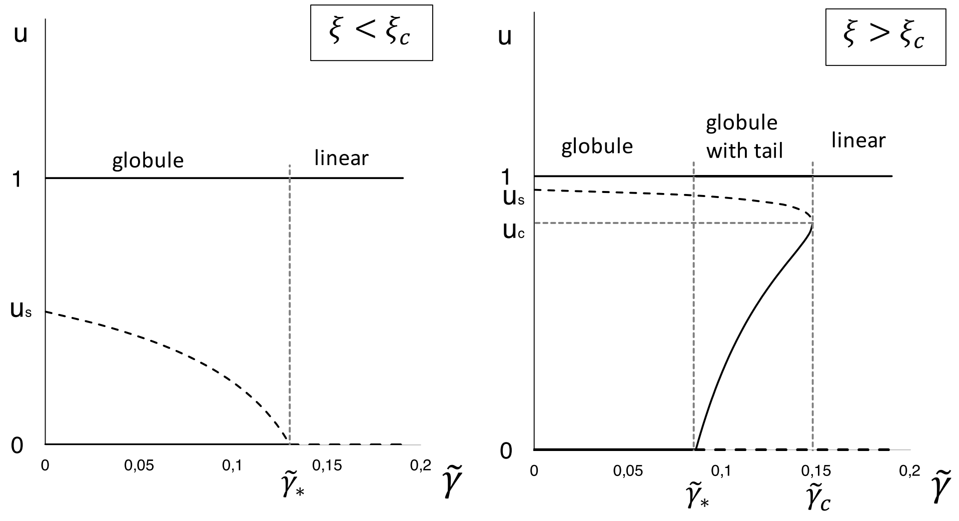

Fig. S3. Bifurcation diagrams of VWF multimer. u is degree of VWF multimer unfolding, i.e. relative length of unfolded part of the molecule. u=0 corresponds to a fully folded globule, u=1 corresponds to a fully unfolded multimer chain. **a**: ξ =2, **b**: ξ =20. Stable stationary states are shown by solid line and unstable states – by dashed line. $\tilde{\gamma}_* = \left(\xi^{1/3} - 1\right)/\xi, \quad \tilde{\gamma}_c = 4/27, \ u_c = 1 - \frac{27}{8\xi}, \ u_s = 1 - \frac{1}{\xi}$.

Another type of transition from the folded to the fully unfolded state takes place for the values of parameter ξ greater than ξ_c. In this case any variation of shear rate $\tilde{\gamma}$ starting in the domain "globule" and finishing in the domain "linear" passes through the "globule with tail" state. This means that under variation of shear rate $\tilde{\gamma}$, an initially folded globule first starts partially to unfold since the boundary $\tilde{\gamma} = \tilde{\gamma}_*(\xi)$ is reached. This looks like a far from equilibrium second-order transition[3,4]. Further increasing of the shear rate $\tilde{\gamma}$ leads to a growth of the length of an unfolded tail (see Fig S3b). If shear rate is increased up to the critical value $\tilde{\gamma} = \tilde{\gamma}_c$, then a fold catastrophe takes place[5,6].

It can be seen from Fig. S3, that under values of parameters belonging to the domains "globule" and "globule with tail", a fully unfolded state (u=1) also exists and is stable with respect to small perturbations. But it may be reached only if shear rate $\tilde{\gamma}$ was initially once increased to the over-critical value $\tilde{\gamma} > \tilde{\gamma}_c$ and then decreases. A fully unfolded state corresponding to the domains "globule" and "globule with tail" cannot be reached by variation of $\tilde{\gamma}$ if $\tilde{\gamma}$ increases from initially small values in the interval $\tilde{\gamma} < \tilde{\gamma}_c$.

Platelet activation diagram derivation

In this part we derive a platelet activation diagram on the $(\xi, \tilde{\gamma})$ parametric plane. We assume the existence of a minimal amount of VWF monomers in multimer

chain that is necessary to activate a platelet. We call this number “minimal platelet activation accord” n_A . In our further consideration, instead of n_A we use for mathematical convenience the parameter $\xi_A = \frac{3}{2} n_A$

It is obvious that if $\xi < \xi_A$, then VWF cannot activate a platelet, no matter what shear rate is applied. The corresponding domain on the platelet activation diagram, Fig. S4, is denoted as “U” (unable to activate).

If $\xi > \xi_A$ and $\tilde{\gamma} > \tilde{\gamma}_c$, i.e. conformational state of VWF corresponds to the domain “linear” on the VWF folding-unfolding diagram (Fig. S2), then VWF is fully unfolded, that means, it can activate a platelet. This area belongs to the upper part of the domain “A” (activate) in the platelet activation diagram (Fig. S4).

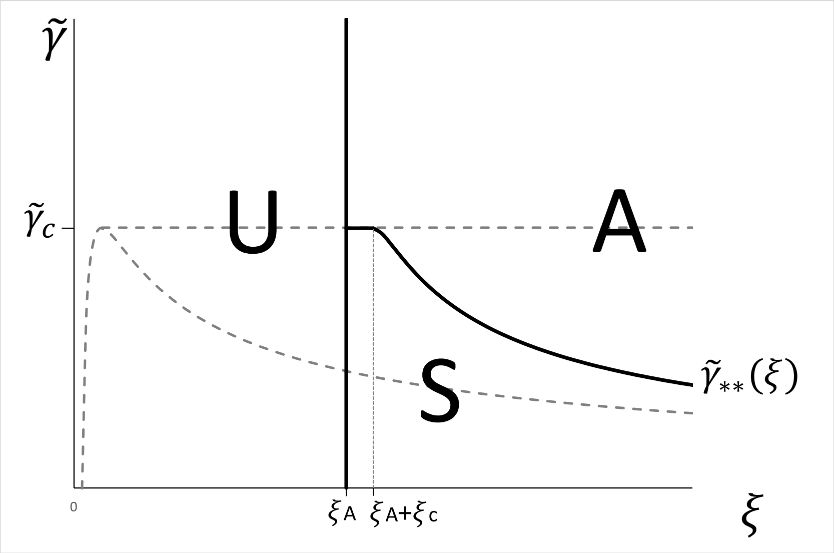

Fig. S4. Platelet activation diagram. Domain “U” corresponds to the situation when VWF is unable to activate platelet ($\xi < \xi_A$). Domain “S” is relevant to subcritical activation of platelets (S14). In this domain VWF can be in one of two different conformational states: one of them is sufficient for platelet activation and the other is not. Symbol “A” denotes the domain corresponding to platelet activation by VWF ($[(\xi_A < \xi < \xi_A + \xi_c) \cap (\tilde{\gamma} > \tilde{\gamma}_c)] \cup [(\xi > \xi_A + \xi_c) \cap (\tilde{\gamma} > \tilde{\gamma}_{**}(\xi))]$).

If $\xi > \xi_A$ but $\tilde{\gamma} < \tilde{\gamma}_c$, then besides a fully unfolded state of VWF multimer, a folded or partially unfolded state exists (see domains “globule” and “globule with tail” in Fig. S2). The possibility of platelet activation by VWF then depends on the value of the VWF unfolded tail length.

This length obviously equals to zero inside the domain “globule” and on the boundary between the domains “globule” and “globule with tail”.

Platelet is activated only if the length of the unfolded tail – un is larger than the minimal platelet activation accord: $un \geq n_A$. (Equivalent to $u\xi \geq \xi_A$).

The substitution of $u\xi = \xi_A$ into the equation (S9) gives the expression:

$$\tilde{\gamma} = \tilde{\gamma}_{**}(\xi) \equiv \frac{(\xi - \xi_A)^{1/3} - 1}{(\xi - \xi_A)} \quad \text{(S12)}$$

For $\xi > \xi_A + \xi_c$ this curve corresponds to the partially unfolded state of the VWF molecule with tail length ξ_A. This means that VWF multimer activates platelets if the values of parameters are within the range that corresponds to the upper part of the domain “globule with tail”:

$$\tilde{\gamma} > \tilde{\gamma}_{**}(\xi), \quad \xi > \xi_A + \xi_c \quad \text{(S13)}$$

(part of domain “A” in Fig. S4).

The domain of the parametric plane denoted as “S” in Fig. S4 corresponds to situations when the platelet may be activated by VWF only if the VWF molecule is fully unfolded. In a partially unfolded state of VWF, relevant to the domain “S”, the tails have subcritical lengths, insufficient for platelet activation ($u\xi < \xi_A$) (see Fig. S4). Domain “S” can be described by the expression:

$$[(\xi_A < \xi < \xi_A + \xi_c) \cap (\tilde{\gamma} < \tilde{\gamma}_c)] \cup [(\xi > \xi_A + \xi_c) \cap \left(\tilde{\gamma} < \frac{(\xi - \xi_A)^{1/3} - 1}{(\xi - \xi_A)}\right)] \qquad \text{(S14)}$$

Transformation of the results from the dimensionless to dimension form

The problem of VWF unfolding is considered mathematically in dimensionless units. For experimental or clinical analysis, the parametric diagram should be converted into a dimension form. Instead of dimensionless multimer length ξ and shear rate $\tilde{\gamma}$ it is necessary to represent the results in usable units.

For instance, the multimer size could be measured in the number of monomers per multimer, *n*. We assume that the width of the multimer chain *d* is of the order of one monomer size (~30 nm). This means that *n=L/d*. Taking in mind that $\xi = \frac{3}{2}\frac{L}{d}$ one has $n = \frac{2}{3}\xi$.

In order to estimate the relation between the dimensionless shear rate $\tilde{\gamma}$ and shear rate $\dot{\gamma}$ measured in sec^{-1}, we take into account that it is usually assumed that the upper value for a critical shear rate inducing a non-reversible platelet aggregation, without any relation to VWF multimer size, is assumed[7,8] to be about 10000 sec^{-1}. We suppose that this corresponds to the critical value of parameter $\tilde{\gamma}$: $\tilde{\gamma}_c = 4/27$, that separates the domains “globule with tail” and “linear” on the parametric plane (Fig. S2). Thus, in order to obtain a proportionality coefficient in the linear dependence between $\dot{\gamma}$ and $\tilde{\gamma}$, a correspondence between $\tilde{\gamma}_c = 4/27$ and $\dot{\gamma}_c = 10000 sec^{-1}$ should be used. As a result, one has: $\dot{\gamma} = \frac{27}{4} \cdot 10^4\ sec^{-1} \cdot \tilde{\gamma} = 6.75 \cdot 10^4\ sec^{-1} \cdot \tilde{\gamma}$ (see Fig. S5).

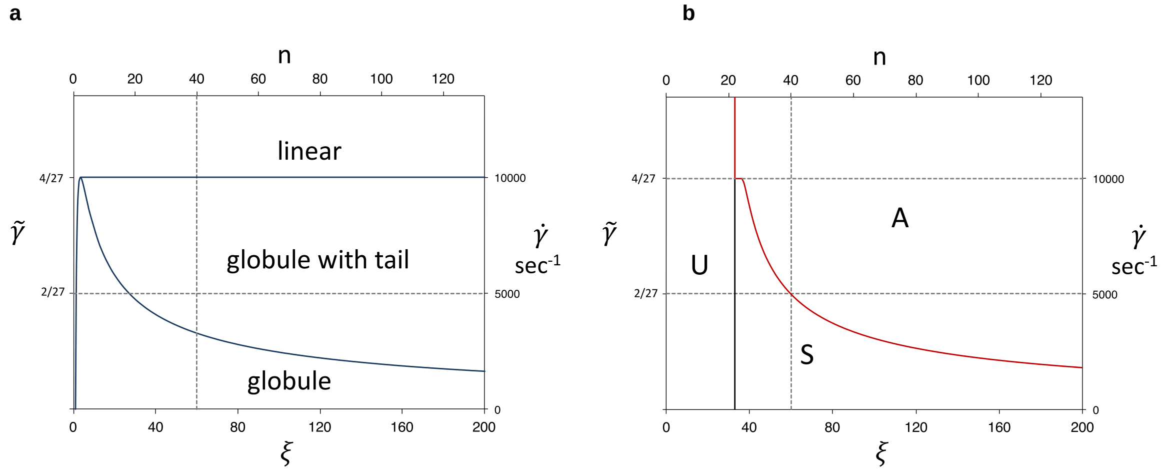

Fig. S5. Parametric diagrams in dimensionless variables and their possible reference to real dimension values. a: VWF folding-unfolding diagram; b: diagram of platelet activation by VWF.

It is clear that these estimates for $\dot{\gamma}_c$ are rough. Quantitative correspondence of dimensionless variables ξ and $\tilde{\gamma}$ to the values of *n* and $\dot{\gamma}$ should be obtained in further

experiments. Probably, further progress in the area could be reached with the aid of optical tweezers method[9,10,11].

Asymptotic formulas for the large VWF molecules

An approach developed here is essentially based on the assumption that a VWF is rather large, so that the concepts of a dense spherical globule and "surface tension" may be introduced.

Certainly, the concept of a spherical globule may not be applied for molecules containing three or less monomeric units ($n \leq 3$) because their compact packaging is possible in one plane. In this sense, a simplest compact 3D packed object may occur only if $n \geq 4$ (starting from tetrahedron).

For this reason, "rigorous formulas" presented in this paper are not applicable for short VWF molecules, consisting of less than 4 monomers. The larger a VWF multimer is, the higher is the correctness of formulas received. Moreover, for practical purposes for large VWF molecules the following asymptotic expressions can be used instead of (S10) and (S12):

$$\tilde{\gamma}_*(\xi) \sim \xi^{-2/3} \quad \text{(S15)}$$

$$\tilde{\gamma}_{**}(\xi) \sim (\xi - \xi_A)^{-2/3} \quad \text{(S16)}$$

Note on the fractal dimensionality of a VWF globule

In this work we considered a VWF multimer as a 3-dimensional object folding into a dense globule. It is very probable that an actual VWF globule is more porous than a macromolecule in the fully condensed 3D model described above. Formally, a "nest-like" model of the object may be described in terms of fractal geometry[12]. Any fractal object may be characterized by fractal dimensionality D.

In this case, the connection between the radius and length of the tail is described as

$$r = \alpha \sqrt[D]{L - x} \quad \text{(S17)}$$

(α is a proportionality coefficient) and supposing that surfaces of globule are measured in power (D-1) of r ($S \sim r^{D-1}$), the equation between the folding and unfolding forces is:

$$1 - \xi^{-\frac{1}{D}}(1-u)^{-\frac{1}{D}} = \tilde{\gamma}\xi^{\frac{D-1}{D}}(1-u)^{\frac{D-1}{D}} \quad \text{(S18)}$$

where $\xi \sim \frac{L}{d}$ and $\tilde{\gamma} \sim \frac{\dot{\gamma}\eta d}{\sigma}$. Analysis of this equation reveals the same 3 states of a D-dimensional globule as in 3D case. The parametric plane $(\xi, \tilde{\gamma})$ is divided into corresponding zones "globule", "globule with tail" and "linear" by the curves:

$$\tilde{\gamma}_* = \frac{\xi^{1/D} - 1}{\xi} \quad \text{and} \quad \tilde{\gamma}_c = \frac{(D-1)^{D-1}}{D^D} \quad \text{for } \xi_c > \left(\frac{D}{D-1}\right)^D \quad \text{(S19)}$$

In the case D=3 this formulas give expressions (S10) and (S11).

Argumentation, similar to that demonstrated above for a 3-dimensional globule, leads to an asymptotic formula for the critical shear rate of platelet activation in a common case of a globule of fractal dimensionality D:

$$\tilde{\gamma}_{**}(\xi) \sim (\xi - \xi_A)^{-(D-1)/D}. \quad \text{(S20)}$$

References:

1. Brochard-Wyart, F., Hervet, H., Pincus, P. Unwinding of Polymer Chains under Forces or Flows. *Europhys. Lett.* **26** (7), 511-516 (1994)

2. De Gennes, P.G. *Scaling Concepts in Polymer Physics* (Cornell University Press, 1979)
3. De Gennes, P.G. Kinetics of collapse for a flexible coil. *J. Physique Lett.* **46**, 639-642 (1985)
4. Lifshitz, I.M., Grosberg, A.Yu. State diagram of a polymer globule and the problem of self-organization of its spatial structure. *Zh. Eksp. Teor. Fiz.* **65**, 2399-2420 (1973) translation from Russian: *Sov. Phys.·JETP* **38** (6) 1198-1208 (1974)
5. Arnold, V.I. *Catastrophe Theory* 3rd ed. (Berlin: Springer-Verlag, 1992)
6. Thom, R. *Structural Stability and Morphogenesis* (Westview Press, 1994)
7. Ruggeri, Z.M., Orje, J.N., Habermann, R., Federici, A.B., Reininger, A.J. Activation-independent platelet adhesion and aggregation under elevated shear stress. *Blood* **108**, 1903-1910 (2006)
8. Themistou, E., *et al.* Application of fluorescence spectroscopy to quantify shear-induced protein conformation change. *Biophysical Journal* **97,** 2567-2576 (2009)
9. Kim, J., Zhang, C., Zhang, X., Springer, T.A. A mechanically stabilized receptor-ligand flex-bond important in the vasculature. *Nature* **466** 992-995 (2010)
10. Ying, J., Ling, Y., Westfield, L.A., Sadler, J.E., Shao, J.Y. Unfolding the A2 domain of von Willebrand factor with the optical trap. *Biophys J.* **98,** 1685–1693 (2010)
11. Arya, M., *et al.* Ultralarge multimers of von Willebrand factor form spontaneous high-strength bonds with the platelet glycoprotein Ib-IX complex: studies using optical tweezers. *Blood* **99,** 3971-3977 (2002)
12. Mandelbrot, B. *The Fractal Geometry of Nature* 1st ed. (W.H. Freeman and Company, 1982)
